# Acute reperfusion intramyocardial hemorrhage leads to regional chronic iron deposition in the heart

**DOI:** 10.1186/1532-429X-15-S1-P174

**Published:** 2013-01-30

**Authors:** Avinash Kali, Ivan Cokic, Andreas Kumar, Sotirios Tsaftaris, Richard L Tang, Matthias G Friedrich, Rohan Dharmakumar

**Affiliations:** 1 Biomedical Imaging Research Institute, Cedars-Sinai Medical Center, Los Angeles, CA, USA; 2 Department of Biomedical Engineering, University of California, Los Angeles, CA, USA; 3 Quebec Heart & Lung Institute, Laval University, Quebec City, QC, Canada; 4 Department of Computer Science and Applications, IMT Institutions, Lucca, Italy; 5 Montréal Heart Institute, Université de Montréal, Montréal, QC, Canada; 6 Stephenson CMR Center, University of Calgary, Calgary, AB, Canada

## Background

Intramyocardial hemorrhage commonly occurs in large reperfused myocardial infarctions. However, its long-term fate remains unexplored. We hypothesized that acute reperfusion intramyocardial hemorrhage leads to chronic iron deposition.

## Methods

Fifteen patients (mean age = 58±8 years; 3 women), who underwent successful angioplasty for first STEMI, were recruited following informed consent. Cardiovascular Magnetic Resonance (CMR) imaging (1.5T) was performed on day 3 and month 6 post-angioplasty. 2D T2* maps (6 TEs = 2.6-13.7 ms; ΔTE=2.2ms) and Late Gadolinium Enhancement (LGE) images of the entire left ventricle (LV) were acquired. Threshold-based image analysis was performed to identify remote, hemorrhagic (Hemo+) and non-hemorrhagic (Hemo-) myocardium.

Fourteen canines, subjected to ischemia-reperfusion (I-R) injury (3 hours of LAD occlusion followed by reperfusion), underwent CMR (1.5T) on days 3 and 56 post-I-R injury. Three sham-operated animals (Shams) were also studied using CMR at similar time points. 2D T2* maps (6 TEs = 3.4-18.4 ms; ΔTE=3.0ms) and LGE images of the entire LV were acquired. Threshold-based image analysis was performed to identify remote, Hemo+ and Hemo- myocardium. Subsequently, animals were euthanized (day 56), hearts were excised and imaged ex-vivo. Sections of Hemo+, Hemo-, remote and Sham myocardium were isolated and histology was performed. The concentration of iron ([Fe]) within each type of tissue was measured using mass spectrometry.

## Results

**Figure 1:**
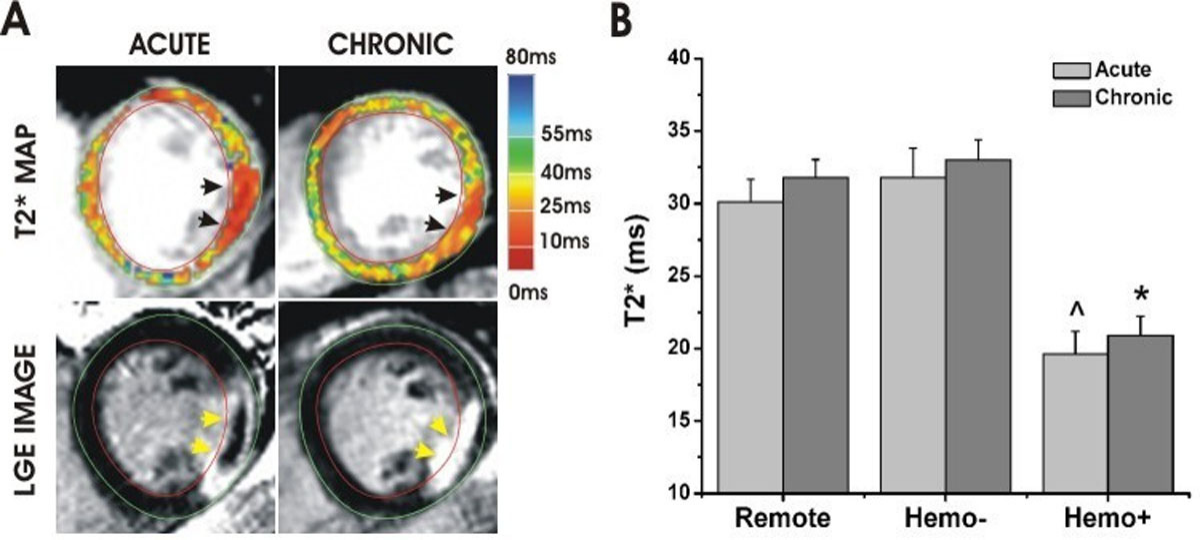
Patient Studies - Representative CMR images (A; acquired from a 42-year old patient following successful angioplasty for first STEMI) with significant T2* loss (arrows) at the site of acute and chronic myocardial infarction (identified by LGE imaging, arrows) are shown. Mean T2* of Hemo+ (B) was 40% lower than that of Hemo- and remote myocardium (p<0.001) on both acute and chronic CMR studies.

Six months post-angioplasty, mean T2* of the scar tissue in patients with Hemo+ infarctions (n=11 as determined by T2* losses within the infarct on day 3 CMR; Figure 1) was 40% lower than that of remote myocardium, suggesting chronic iron deposition (p<0.001). In contrast, mean T2* of Hemo- infarctions (n=4) was not significantly different from that of remote myocardium at both 3 days and 6 months post-angioplasty (p=0.51).

**Figure 2:**
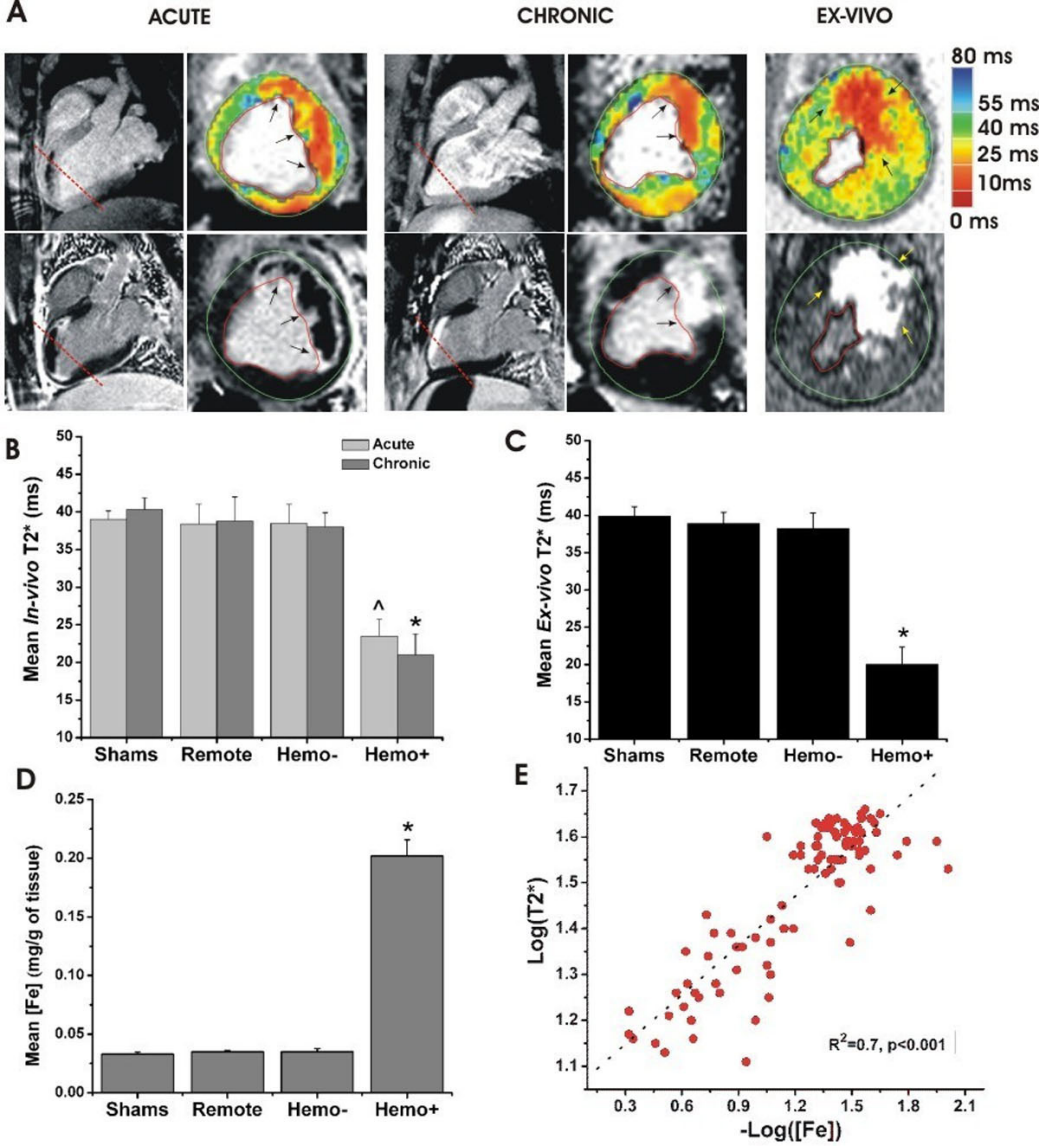
Animal Studies -Representative CMR images (A; T2* in the top row and LGE in the bottom row) acquired from an animal with hemorrhagic myocardial infarction in acute and chronic phases along the long- and short-axis (along the dashed red line in the long-axis images), together with corresponding ex-vivo images are shown (A). In-vivo T2* images (both acute and chronic phases) clearly demonstrate the evidence of signal loss in the LAD territory. Both mean in-vivo T2* (B) and ex-vivo T2* (C) of Hemo+ sections were 40% lower than those of Shams, Remote, and Hemo- in both acute and chronic phases (p<0.001). Mass spectrometric analysis (D) showed that iron content of Hemo+ tissue was 10-fold higher than that of other tissues (p<0.001). Linear regression analysis (E) between log(ex-vivo T2*) and -log([Fe]) showed a strong correlation (R2 = 0.74; p<0.001).

In canines, in-vivo mean T2* of Hemo+ myocardium was 40% lower than those of Sham, remote and Hemo- myocardium (p<0.001) at both 3 days and 56 days post-I-R injury (Figure 2B). Similarly, mean ex-vivo T2* of Hemo+ myocardium was 40% lower than those of Sham, remote and Hemo- myocardium (p<0.001; Figure 2C). Perl's stain confirmed localized chronic iron deposition only within Hemo+ infarctions. Mean [Fe] of Hemo+ infarctions was nearly 10-fold higher than those of Sham, remote and Hemo- myocardium (p<0.001; Figure 2D). A strong linear relationship was observed between log(ex-vivo T2*) and -log([Fe]) (R^2^=0.7; p<0.001; Figure 2E).

## Conclusions

Acute reperfusion intramyocardial hemorrhage leads to regional chronic iron deposition within the infarct zones. T2* CMR can accurately characterize localized chronic iron deposition following reperfusion-induced myocardial hemorrhage. The clinical significance of this finding remains to be investigated.

## Funding

This work was supported in part by grants from American Heart Association (SDG 0735099N) and National Heart, Lung, And Blood Institute (HL091989).

